# Multi-Core-Fiber-Enabled Integrated Communication and Discrete Sensing in Smart Cable Systems: Experimental Demonstration

**DOI:** 10.3390/s26196018

**Published:** 2026-09-23

**Authors:** Xinyi Xie, Hao Wu, Yucheng Fan, Yixin Gao, Songyi Zhang, Guoxiang Xu, Cheng Yang, Siyuan Wang, Rendong Xu, Biao Huang

**Affiliations:** 1 School of Intelligent Manufacturing Ecosystem, Xi’an Jiaotong-Liverpool University, Suzhou 215400, China; xinyi.xie@xjtlu.edu.cn (X.X.); hao.wu25@student.xjtlu.edu.cn (H.W.); songyi.zhang24@student.xjtlu.edu.cn (S.Z.); biao.huang@xjtlu.edu.cn (B.H.); 2 College of Intelligent Manufacturing, Suzhou Centennial College, Suzhou 215123, China; fanyc@scc.edu.cn; 3 Department of Aeronautical and Aviation Engineering, The Hong Kong Polytechnic University, Hong Kong, China; yixin.gao@connect.polyu.hk; 4 Jiangsu Key Laboratory for Undersea Communications and Sensing, Suzhou 215163, China; xugx@hmntech.com (G.X.); cheng.yang@hmntech.com (C.Y.); wangsiyuan@hmntech.com (S.W.)

**Keywords:** smart cable, multi-core fiber, space-division multiplexing, integrated sensing and communication, discrete sensing, sensing-data backhaul

## Abstract

Smart cables extend submarine communication infrastructure toward marine environmental sensing and sensing-data backhaul. This paper investigates a multi-core fiber (MCF)-based prototype in which high-speed communication traffic and discrete-sensor data are routed through different spatial cores rather than sharing the same optical core. A 100 km weakly coupled four-core MCF was characterized and used as the central spatial-routing section, with two cores assigned to bidirectional communication and two cores to bidirectional sensing-data backhaul. At 1625 nm, the measured inter-core crosstalk (IC-XT) values for the four reported measurements were −53.1, −52.6, −51.2, and −53.7 dB/100 km. The same MCF section was incorporated into repeatered and unrepeatered hybrid laboratory configurations, with additional single-mode-fiber spans extending the total link lengths to 500 km and 300 km, respectively. Under the tested 400 Gbps condition, no frame loss or system alarm was observed during 17 h of operation; the round-trip delay was approximately 2.35 ms and the packet jitter remained below 0.3μs. The sensing-node velocity channels recorded transient vibration responses and long-duration spectral distributions consistent with those of a reference CDS-E20 seismometer. These results support the feasibility of spatial-core routing for MCF-based smart cable prototypes.

## 1. Introduction

In 2010, You [1] discussed reusing telecommunication cables for scientific monitoring, and the Scientific Monitoring and Reliable Telecommunications (SMART) cable concept has since been developed through community efforts, engineering reviews, and standardization activities [2,3]. Recent workshop and standardization documents further emphasize that reliability, power feeding, maintainability, and data backhaul must be considered together when integrating environmental sensors into submarine cable systems [4,5]. Such infrastructure is attractive for marine observation because submarine communication cables provide wide spatial coverage in ocean regions where conventional observation networks are difficult and expensive to deploy.

Current marine engineering studies have moved this concept from a broad vision toward cable demonstrators and sensor-integrated systems. In 2025, Matos et al. integrated pressure sensors into a smart marine cable demonstrator for wave and tidal observations, showing the potential of marine cables as real-time ocean monitoring platforms [6]. A Sensors review of seismic monitoring based on submarine optical cables summarized interferometric, fiber-Bragg-grating, polarimetric, and distributed acoustic sensing routes and their relevance to submarine seismology [7]. These works position smart cable research as both a communication and a marine engineering problem involving sensing access, environmental coupling, long-term reliability, and data return to shore. Related studies in other harsh environments further indicate that the long-term performance of sensing-enabled optical cables can be affected by material–environment interactions. Perrot et al. investigated optical-fiber cables for underground nuclear-waste monitoring and reported degradation associated with radiation exposure and hydrogen diffusion from cable materials [8].

Optical-fiber infrastructure is also being extended to other multifunctional applications that combine communication with remote power delivery. Diouf et al. demonstrated simultaneous power-over-fiber (PoF) and communication over a single-mode link for extending cabled seafloor observatories [9]. Multicore fibers have likewise been used to spatially combine PoF with communication or radio-over-fiber functions, including a 10 km MCF PoF/5G-fronthaul link [10] and a radio-and-power-over-fiber demonstration using multicore fiber [11]. These examples show that spatial channels can be used not only for capacity scaling but also for functional integration in optical infrastructure.

A central issue in smart cable systems is how sensing information can coexist with high-speed communication services. Discrete electronic sensors, such as pressure, temperature, acceleration, acoustic, or seismic sensors, usually generate low-rate monitoring data with modest bandwidth requirements. If sensing data are optically transmitted through the same physical core as high-capacity communication traffic, coexistence must be managed through wavelength allocation, spectrum placement, modulation design, or receiver processing. These approaches are technically effective, but the sensing and communication functions remain coupled through a shared optical path and therefore require careful control of optical power, spectrum occupancy, nonlinear interaction, and signal-processing compatibility. In submarine communication systems, preserving the wavelength and spectral resources of the traffic-carrying cores is also an important engineering consideration. A spatial-domain implementation therefore offers a complementary option in which sensing-data backhaul is assigned to dedicated spatial cores rather than reserving wavelength or spectral resources within the communication cores, while the functions remain integrated within a common fiber cladding.

Several single-core integrated sensing and communication studies have addressed this coexistence problem from the signal domain. In 2025, Cai et al. proposed a dense and low-interference SMART-network scheme based on digital subcarrier modulation (DSCM). Electronic-sensor data were converted into frequency-modulated electrical signals and optically placed in spectral gaps between communication subcarriers, improving spectral utilization while maintaining compatibility with coherent-communication digital signal processing (DSP) [12]. In a related study, Wu et al. used frequency modulation and optical single-sideband modulation to place multiple sensing subcarriers close to the communication spectrum without direct spectral overlap, allowing sensing information to be recovered using processing already available in a coherent receiver [13]. Forward-transmission distributed sensing provides another form of signal-level integration. Zhu et al. combined a 150 Mb/s on–off-keying communication system with forward-transmission distributed vibration sensing over approximately 101 km of single-mode fiber while evaluating the mutual influence of the two functions [14]. These studies establish signal-domain and shared-fiber routes for smart cable coexistence through spectral allocation, optical modulation, communication-compatible DSP, and forward-transmission sensing.

Distributed fiber sensing is another important approach to observing submarine cables. Polarization-based cable sensing, ultrastable interferometry, and distributed acoustic sensing have demonstrated substantial scientific value in seismology and water-wave monitoring [15,16]. Forward-transmission sensing and fiber communication/sensing integration have also been investigated as complementary approaches for long optical links [17,18]. Chen et al. demonstrated long-range distributed vibration sensing using phase-sensitive forward optical transmission [19], subsequently extended the approach to super-long-range sensing based on polarimetric forward transmission [20], and further integrated polarimetric forward-transmission communication with distributed sensing in a long-distance single-fiber system [21]. Recent coexistence studies and ocean-oriented reviews further illustrate the engineering relevance of distributed acoustic sensing in communication-fiber systems [22,23]. These approaches use the optical fiber itself as the sensing medium. By contrast, the present architecture uses discrete electronic sensing nodes and MCF spatial cores to return their data. The two routes therefore address different but complementary sensing functions.

The present paper explores this complementary spatial-domain route. A characterized 100 km weakly coupled four-core MCF is used as the central spatial-routing section of the smart cable prototype. Two cores form the bidirectional communication path, while the other two cores form the bidirectional sensing-data-backhaul path. This arrangement avoids direct sharing of the same optical core and avoids reserving sensing wavelengths within the communication cores, while retaining the compact common-cladding structure of MCF. The corresponding engineering trade-offs are the need for fan-in/fan-out spatial access and finite inter-core crosstalk, both of which are explicitly considered in the prototype. This engineering motivation is specific to the architecture evaluated here and is not presented as a claim of universal quantitative superiority over same-core coexistence or multi-SMF implementations. Additional single-mode-fiber spans extend the total laboratory link lengths for long-distance repeatered and unrepeatered validation.

Multi-core fiber has been widely studied as a space-division-multiplexing medium for optical communication capacity scaling [24,25]. Its relevance to this work is the availability of multiple spatial channels inside one cladding. Field trials of communication and sensing in space-division-multiplexing optical fiber cables have shown that spatial channels can support new system-level combinations of communication and sensing [26]. A recent field demonstration further reported real-time high-capacity communication and sensing over a deployed multi-core fiber cable [27]. For smart cable systems using discrete sensing nodes, this spatial degree of freedom can be used as a physical routing mechanism for sensing-data backhaul.

Based on this motivation, this paper presents an architectural design and prototype demonstration of an MCF-based smart cable system. The main contributions are as follows. First, we implement a 100 km weakly coupled four-core MCF-based smart cable architecture, with two cores assigned to communication and two to sensing-data backhaul. Second, a spatial-core-routing and hybrid-link design is established for both repeatered and unrepeatered configurations while keeping the MCF section as the functional spatial-routing medium. Third, the architecture is validated in 500 km repeatered and 300 km unrepeatered laboratory links through 400 Gbps communication testing and a comparative velocity-response evaluation against a reference seismometer.

## 2. Theoretical Basis

This section summarizes the physical basis of the prototype, including same-core nonlinear interaction, spatial-core assignment in weakly coupled MCF, inter-core crosstalk, node access loss, and link power budget. The purpose is to connect the system architecture to measurable optical quantities used later in the experimental characterization.

### 2.1. Smart Cable Coexistence Challenge

When communication and sensing-related optical signals propagate in the same core, the Kerr nonlinearity can couple their optical phases through self- and cross-phase modulation [28]. For a simplified two-wave interaction, the nonlinear phase accumulated by the sensing-related signal can be represented as

(1) $$\Phi_{\mathrm{NL}}\left(t\right)=\gamma L_{\mathrm{eff}}\left[P_{\mathrm{sens}}\left(t\right)+2P_{\mathrm{com}}\left(t-\tau \right)\right],$$

where γ is the nonlinear coefficient, $L_{\mathrm{eff}}$ is the effective interaction length, $P_{\mathrm{sens}}$ and $P_{\mathrm{com}}$ are the instantaneous optical powers of the sensing-related and communication signals, respectively, and τ represents their relative group-delay walk-off. This expression motivates a spatial-domain implementation in which the two data types do not intentionally share the same core.

### 2.2. MCF Spatial Multiplexing and Core Assignment

A multi-core fiber introduces several spatial channels within one cladding. In a weakly coupled MCF, the core geometry and refractive-index profile are selected so that each core behaves predominantly as an independent transmission path, although finite coupling remains. This property allows different functions to be assigned to different cores without requiring the functions to occupy the same optical channel.

The prototype uses four spatial cores. Two cores form the two propagation directions of the communication path, and the other two cores form the two propagation directions of the sensing-data-backhaul path. The architecture is therefore described as spatial routing or physical separation rather than absolute isolation: finite inter-core crosstalk, fan-in/fan-out loss, splice loss, and component imperfections remain relevant engineering quantities.

### 2.3. Inter-Core Crosstalk

Inter-core crosstalk (IC-XT) quantifies optical power coupled from an excited core into another monitored core. We formulate the IC-XT power ratio as follows [29]:

(2) $$XT_{\mathrm{dB}}=10\log_{10}\left(\frac{P_{\mathrm{XT}}}{P_{s}}\right),$$

where $P_{\mathrm{XT}}$ is the leakage power observed in the monitored core, and $P_{s}$ is the corresponding signal-power reference. More-negative values therefore indicate weaker inter-core leakage.

Previous studies have shown that IC-XT can influence transmission performance in weakly coupled MCF systems, with the resulting penalty depending on transmission distance, modulation format, and operating conditions [29,30,31]. Accordingly, we treat IC-XT as a directly measured fiber parameter, while we evaluate link-level communication performance experimentally in Section 5.1. Section 4.1 reports the fiber-level measurements.

### 2.4. Node Loss and Power Budget

The sensing-node access design relies on fan-out and fan-in devices to separate and recombine the spatial cores. Their insertion losses, together with splice and connector losses, must be included in the link budget. The node-related loss is defined as:

(3) $$L_{\mathrm{node}}=L_{\mathrm{fanout}}+L_{\mathrm{fanin}}+L_{\mathrm{splice}},$$

where $L_{\mathrm{fanout}}$ and $L_{\mathrm{fanin}}$ are the insertion losses of the fan-out and fan-in devices, respectively, and $L_{\mathrm{splice}}$ is the splice loss associated with the node access path.

For a link containing optical amplification, the received power can be represented by

(4) $$P_{\mathrm{rx}}=P_{\mathrm{tx}}-\alpha L-L_{\mathrm{node}}-L_{\mathrm{conn}}+\sum_{k=1}^{N_{\mathrm{amp}}}G_{k},$$

where $P_{\mathrm{tx}}$ is the launch power, α is the fiber attenuation coefficient, *L* is the total fiber length, $L_{\mathrm{conn}}$ is the aggregate connector loss, $G_{k}$ is the optical gain of the *k*-th amplifier, and $N_{\mathrm{amp}}$ is the number of amplifying stages. This expression connects the component-level losses to the repeatered laboratory configuration; the measured received optical power and OSNR are reported with the communication experiment in Section 4.3.

## 3. System Architecture

### 3.1. Overall Architecture

The proposed system consists of shore-side control and communication functions, an MCF spatial-routing section, spatial access devices, and underwater sensing nodes. The shore-side functions include the 400 Gbps communication terminal, timing service, host switches, network management, and power-feeding equipment. The underwater domain contains the four-core MCF section, fan-in/fan-out access, sensing nodes, and either repeatered or unrepeatered transmission sections.

Figure 1a presents the application-level marine scenario, in which two cable landing stations are connected through the submarine link and the sensing nodes return monitoring information to shore-side and cloud services. Figure 1b maps the concept to the 500 km repeatered architecture, where sensing nodes are arranged independently of the communication repeaters. Figure 1c shows the corresponding 300 km unrepeatered architecture without in-line communication repeaters. In these schematics, SLTE denotes Submarine Line Terminal Equipment, SLM denotes Submarine Line Monitor, ODF denotes Optical Distribution Frame, CTC denotes Cable Termination Cabinet, BMH denotes Beach Manhole, NMS denotes Network Management System, and PFE denotes Power Feeding Equipment.

### 3.2. Core Assignment and Sensing Node Access

Within the four-core MCF, Core 1 and Core 2 form the bidirectional communication pair, with one core used for each propagation direction. The communication pair is used for real-time transmission and is not configured as a redundant pair. Core 3 and Core 4 form the sensing-data-backhaul pair in opposite directions, allowing sensing-node data to be returned toward both shore ends and providing dual-end data availability. This assignment avoids intentional sharing of the communication cores by the sensing-data streams.

After fan-out, the communication cores continue through the communication transmission path, while the sensing-data cores connect to the sensing-node access path. After optoelectronic processing and data aggregation at the sensing nodes, the sensing data are transmitted toward the shore-side host switches through the sensing backhaul cores. The corresponding fan-in devices remap the optical paths into the MCF. Figure 2 illustrates this functional assignment.

The shore-side blocks in Figure 2 follow the same terminology as Figure 1: SLTE is the Submarine Line Terminal Equipment, SLM is the Submarine Line Monitor, ODF is the Optical Distribution Frame, and CTC is the Cable Termination Cabinet. These blocks represent the functional interfaces between the spatially routed optical paths and the shore-side communication, monitoring, and power systems.

### 3.3. Repeatered and Unrepeatered Configurations

The same spatial-routing principle can be incorporated into repeatered and unrepeatered smart cable configurations. In a repeatered architecture, sensing nodes can be implemented as independent functional nodes on the sensing-data path while the communication path remains associated with its conventional amplification/repeater functions. This arrangement avoids requiring the sensing function to share the same optical core or signal-processing channel as the communication traffic.

Both laboratory configurations use the same 100 km four-core MCF as the functional spatial-routing section. In the repeatered experiment, additional standard G.654.D ultra-low-loss single-mode-fiber (SMF) spans and optical amplification extend the complete laboratory link to 500 km. In the unrepeatered experiment, additional G.654.D SMF sections extend the total link to 300 km without in-line optical amplification. In both cases, spatial-core routing is implemented in the 100 km MCF section.

## 4. Experimental Setup

### 4.1. Characterization of the 100 km Four-Core MCF

The system-level prototype employed a 100 km weakly coupled four-core MCF section that was designed and fabricated for this work. The optical parameters reported in this subsection were obtained from the final fiber inspection record and represent fiber-level characterization before the cable-forming processes; process-level manufacturing details are outside the scope of the present system study. Figure 3 provides the physical context of the four-core fiber and its intended marine-cable integration. Figure 3a shows a representative cable cross-sectional arrangement, while Figure 3b shows the end face of the four-core MCF used for spatial routing.

Only parameters directly recorded in the final fiber inspection record and relevant to the present prototype are reported here. The measured cladding diameter was 124.73 μm. The adjacent-core spacings for Core 1–2, Core 2–3, Core 3–4, and Core 4–1 were 42.798, 42.981, 42.815, and 42.833 μm, respectively, while the opposite-core spacings for Core 1–3 and Core 2–4 were 60.550 and 61.667 μm. The inspection record lists a PK8000 optical time-domain reflectometer (Photon Kinetics, Inc., Beaverton, OR, USA) for attenuation characterization, a PK2800 fiber-dispersion tester (Photon Kinetics, Inc., Beaverton, OR, USA) for chromatic-dispersion characterization, and an FSM-100P+ direct core-to-core crosstalk tester (Fujikura Ltd., Tokyo, Japan) for the IC-XT measurement.

The optical and transmission parameters used in the present characterization are summarized in Table 1. At 1550 nm, the attenuation coefficient was 0.182 dB/km for each of the four reported core measurements. The measured mode-field diameters were 10.73, 10.84, 10.79, and 10.72 μm, the cutoff wavelengths were 1443.25, 1448.56, 1443.47, and 1448.32 nm, and the chromatic-dispersion coefficients were 20.368, 20.377, 20.378, and 20.390 ps/(nm·km). The numerical aperture was 0.12, and the reported group refractive indices were 1.4683 at 1550 nm and 1.4684 at 1625 nm. The same inspection record marks the dispersion spectrum over 1310–1625 nm as compliant but does not tabulate separate per-core numerical attenuation or dispersion values at 1310 nm; no unsupported 1310-nm values are therefore introduced here. The quantitative values emphasized in this study correspond to the C-band communication operation and to the wavelengths at which the final inspection record provides numerical measurements.

Inter-core crosstalk was characterized at 1625 nm using the direct core-to-core test method recorded for the FSM-100P+ system. The four reported IC-XT values were −53.1, −52.6, −51.2, and −53.7 dB/100 km, ranging from −53.7 to −51.2 dB/100 km. These are the four values reported in the final inspection record; the record does not provide a separate full pairwise crosstalk matrix. The inspection record also reports 1625-nm macrobending losses of 0.05, 0.02, 0.06, and 0.04 dB under a Φ30 mm × 10-turn condition. These values are treated as directly measured fiber parameters; link-level communication performance is evaluated independently in Section 5.1.

### 4.2. Laboratory Testbed

A prototype testbed was established to evaluate simultaneous 400 Gbps communication transmission and discrete-sensor data backhaul. The system includes the four-core-MCF access section, fan-in/fan-out devices, communication transmission and test equipment, optical amplifiers, a time server, sensing nodes, shore-side host switches, remote power-supply equipment, and a reference seismometer. Figure 4 shows the laboratory realization of these functional blocks.

Figure 4a corresponds to the communication-terminal and long-distance optical-link functions introduced in Figure 1. The 400 Gbps coherent transmission platform and tester implement the shore-side communication and monitoring functions, while the optical amplifiers and fiber spools form the long-distance optical path. Figure 4b realizes the sensing-data path: the sensing nodes acquire vibration data, the two host switches at the opposite ends receive the returned sensing data, and the timing server provides the common time reference. These host-switch and timing functions correspond directly to the HOST SWITCH and TIME SERVICE blocks in the system architecture. The CDS-E20 serves as the laboratory reference instrument for sensing-response comparison.

### 4.3. Laboratory Link Configurations and Communication Measurements

The same core assignment was used in both long-distance configurations. Core 1 and Core 2 carried the two directions of the 400 Gbps communication link, while Core 3 and Core 4 carried the opposite-direction sensing-data-backhaul paths. For readability, Figure 5 and Figure 6 group each transmit/receive pair into a single line marked T/R; the T/R notation therefore represents two directional core paths rather than a single physical core. In the schematics, ZXR10 denotes the 400 Gbps coherent transceiver platform (ZTE Corporation, Shenzhen, China; provided by Jiangsu Future Network Group Co., Ltd., Nanjing, China), SW denotes the sensing-data host switch, and FI/FO denote the fan-in/fan-out devices.

For the repeatered configuration, the 100 km four-core MCF spatial-routing section was combined with additional G.654.D SMF spans to form a total 500 km optical path. The blocks labeled “Repeater” in the laboratory schematic were implemented with conventional terrestrial optical-fiber amplifiers that provided optical gain only; no optoelectronic 3R regeneration was employed. Figure 5 shows the resulting laboratory topology.

For the unrepeatered configuration, the same 100 km four-core MCF section was combined with additional G.654.D SMF trunk/branch spans to form a total 300 km link without in-line optical amplification. Figure 6 shows this arrangement. The additional SMF sections extend the laboratory transmission distance, whereas the 100 km MCF remains the section that implements communication/sensing-data spatial routing.

The coherent communication transmitter operated on ITU channel CH26 at 1538.98 nm. The communication launch power was approximately 8 dBm, and the received optical power at the input of the 400 Gbps module was controlled at approximately −10 dBm in both configurations. The measured OSNR was approximately 26 dB for the 500 km repeatered link and 22 dB for the 300 km unrepeatered link. The sensing-backhaul optical modules operated within the C-band (1530–1565 nm).

For the communication stability test, 16 Internet Protocol test streams of 25 Gbps each produced an aggregate traffic rate of 400 Gbps. The monitored quantities were round-trip delay (RTD), packet jitter, current frame-loss count, and system alarms. The monitoring period was 17 h.

### 4.4. Sensor Performance Measurements

The sensing comparison uses only the velocity-sensing channel of the prototype sensing node. The selected velocity sensor has a nominal response range of 1–150 Hz, a nominal sensitivity of 500 V/(m/s), and a specified sensitivity tolerance of +5%/–10%.

One CDS-E20 reference seismometer (Institute of Geology and Geophysics, Chinese Academy of Sciences, Beijing, China) was used in the comparison and was placed adjacent to the sensing node. The CDS-E20 reference seismometer has a manufacturer-specified nominal operating band of 20 s–150 Hz and was used to provide the reference three-component velocity records for the sensing comparison. The two records were acquired using the laboratory timing infrastructure, but no independent end-to-end hardware synchronization-accuracy test was performed.

The sensing-node velocity data were originally sampled at 250 Hz and were resampled to 100 Hz for comparison. The comparative analysis was restricted to the 0.5–20 Hz post-processing band; this analysis band should not be interpreted as the calibrated end-to-end nominal frequency response of the integrated sensing node. The two data streams were de-meaned and converted from counts to velocity using the fixed processing coefficients used in the analysis pipeline, approximately $8.472\times 10^{8}$ counts/(m/s) for the sensing-node velocity channel and $1.714\times 10^{9}$ counts/(m/s) for the CDS-E20. A complete frequency-dependent instrument response was not available for the present analysis, and no frequency-dependent response deconvolution was therefore applied. Channel pairing and polarity were determined in post-processing by testing the three-component combinations and selecting the pairing with the highest time-domain Pearson correlation. The resulting pairings used for the time-domain comparison are V2/BHE, V3/BHN, and V1/BHZ. After resampling, no additional sample-level temporal shift was applied; peak normalization was used only for waveform visualization.

For the time-domain comparison, the correlation coefficient for two aligned time series, $x_{n}$ and $y_{n}$, is defined as:

(5) $$R_{t}=\frac{\sum_{n}\left(x_{n}-\bar{x}\right)\left(y_{n}-\bar{y}\right)}{\sqrt{\sum_{n}{\left(x_{n}-\bar{x}\right)}^{2}}\sqrt{\sum_{n}{\left(y_{n}-\bar{y}\right)}^{2}}},$$

where $\bar{x}$ and $\bar{y}$ are the sample means over the selected comparison window.

The waveform comparison uses a representative 30 s segment starting at 14 September 2025 01:23:10 UTC from the long-duration laboratory record. A clear transient was recorded simultaneously by both instruments, with the principal event occurring at approximately 9–11 s within the segment. The event was an ambient machinery-induced transient captured during routine operation of large equipment in the laboratory environment.

Long-duration spectral behavior was evaluated independently using ObsPy (v1.5.0) probabilistic power spectral density (PPSD) implementation [32]. The available common record from 12 September 2025 02:21:56.051700 UTC to 15 September 2025 00:40:09.098300 UTC was processed using 60 s PPSD windows with 50% overlap. Valid 60 s segments were accumulated to obtain the probability distribution of spectral power. The standard seismic noise models provided by ObsPy are included as contextual spectral references for the laboratory measurements.

Because the present campaign did not include a complete instrument-level calibration, intrinsic self-noise, end-to-end dynamic range/full-scale input, absolute hardware synchronization accuracy, controlled repeatability, and node-to-node variability are not reported. These quantities require dedicated calibration or repeated-excitation measurements and are reserved for future instrument-level characterization. The present sensing analysis is therefore limited to the velocity-channel response-consistency measurements described above.

## 5. Results and Discussion

### 5.1. Communication Performance

The 400 Gbps communication link remained stable throughout the 17 h monitoring period. Figure 7 presents the monitored indicators at the communication carrier wavelength of 1538.98 nm (CH26).

Figure 7a shows an RTD of approximately 2.35 ms; RTD denotes the elapsed time for the transmitted test data to propagate through the link and return to the measurement endpoint. Figure 7b shows that the packet jitter of the 16 test streams remained below 0.3μs. Figure 7c reports the instrument’s current frame-loss count, for which no non-zero value was observed in the monitored trace. No system alarm was observed during the 17 h test. Together with the measured OSNR values of approximately 26 dB and 22 dB for the repeatered and unrepeatered configurations, respectively, these results confirm stable 400 Gbps operation under the tested laboratory conditions. The communication metrics reported here correspond to the simultaneous communication and sensing-data-backhaul configuration used in the prototype. Because the campaign did not include a matched communication-only baseline or a same-core wavelength/spectrum-sharing reference configuration, the present data do not quantify an on/off coexistence penalty or a relative improvement over those alternative implementations.

### 5.2. Sensing Response Consistency

Figure 8 compares the selected 30 s transient velocity record from the sensing-node velocity channels and the CDS-E20 velocity channels. Figure 8a–c show the V2/BHE, V3/BHN, and V1/BHZ waveform pairs, respectively, and the $R_{t}$ values quantify their time-domain Pearson correlation after the processing described in Section 4.4. Figure 8d places the selected 30 s segment within the long-duration record. The common transient around 9–11 s is visible in both instruments, supporting consistent capture of the same velocity response.

To examine the spectral behavior over a much longer interval, Figure 9 presents PPSD distributions calculated from the common velocity record between 12 September 2025 02:21:56.051700 UTC and 15 September 2025 00:40:09.098300 UTC. Figure 9a,c,e show the sensing-node velocity channels V1, V2, and V3, respectively, whereas Figure 9b,d,f show the CDS-E20 BHE, BHN, and BHZ velocity components. The color scale represents the occurrence probability of spectral-power values accumulated from the valid 60 s windows. The black/gray reference curves are the standard seismic noise models supplied by ObsPy and are included as contextual references rather than as acceptance bounds for the laboratory environment. The sensing-node PPSD represents the total recorded laboratory background under the test conditions, including environmental, installation, and system contributions; it is not interpreted as the intrinsic self-noise of the sensing node.

The time-domain and long-duration PPSD results provide complementary evidence. The transient comparison verifies that the sensing-node and CDS-E20 velocity channels respond consistently to the same laboratory event, while the PPSD comparison shows their longer-term velocity-spectral behavior over multiple days. Together, these measurements demonstrate that vibration-velocity data acquired by the sensing node can be returned through the proposed MCF-based sensing-data-backhaul path while retaining consistent time-domain and long-duration spectral characteristics. The sensing analysis is therefore interpreted as a prototype-level velocity-response-consistency assessment under ambient laboratory vibration rather than a complete instrument-level metrological calibration.

## 6. Conclusions

This paper presents an architectural design and prototype demonstration of an MCF-based smart cable system. A characterized 100 km weakly coupled four-core MCF served as the spatial-routing section, with two cores forming the bidirectional 400 Gbps communication path and two cores forming the bidirectional sensing-data-backhaul path. The final fiber inspection record reported IC-XT values of −53.1, −52.6, −51.2, and −53.7 dB/100 km at 1625 nm. The same MCF section was incorporated into 500 km repeatered and 300 km unrepeatered hybrid laboratory links using additional G.654.D SMF spans.

During 17 h of 400 Gbps operation, the monitored trace showed no system alarms or non-zero frame-loss values. The RTD was approximately 2.35 ms, packet jitter remained below 0.3μs, and the measured OSNR was approximately 26 dB and 22 dB for the repeatered and unrepeatered configurations, respectively. On the sensing side, the prototype validation compared only the sensing-node velocity channels with the corresponding CDS-E20 velocity channels. A representative transient event showed consistent three-component time-domain velocity responses, while long-duration PPSD analysis provided a multi-day spectral comparison. These results support the preliminary feasibility of spatial-core routing as a practical method for combining high-speed communication with discrete-sensor data backhaul in a smart cable prototype; they are not interpreted as a quantitative superiority claim over untested same-core or multi-SMF baselines.

Future work will focus on engineering the integration of the current prototype into a compact smart sensing box, including high-density sensing electronics, watertight and pressure-resistant packaging, corrosion-resistant structural design, and cable-level mechanical integration. Longer-term underwater deployment and validation using an ocean-bottom seismometer as a reference instrument will assess sensing performance under representative marine conditions and support multi-source data interpretation.

## Figures and Tables

**Figure 1:**
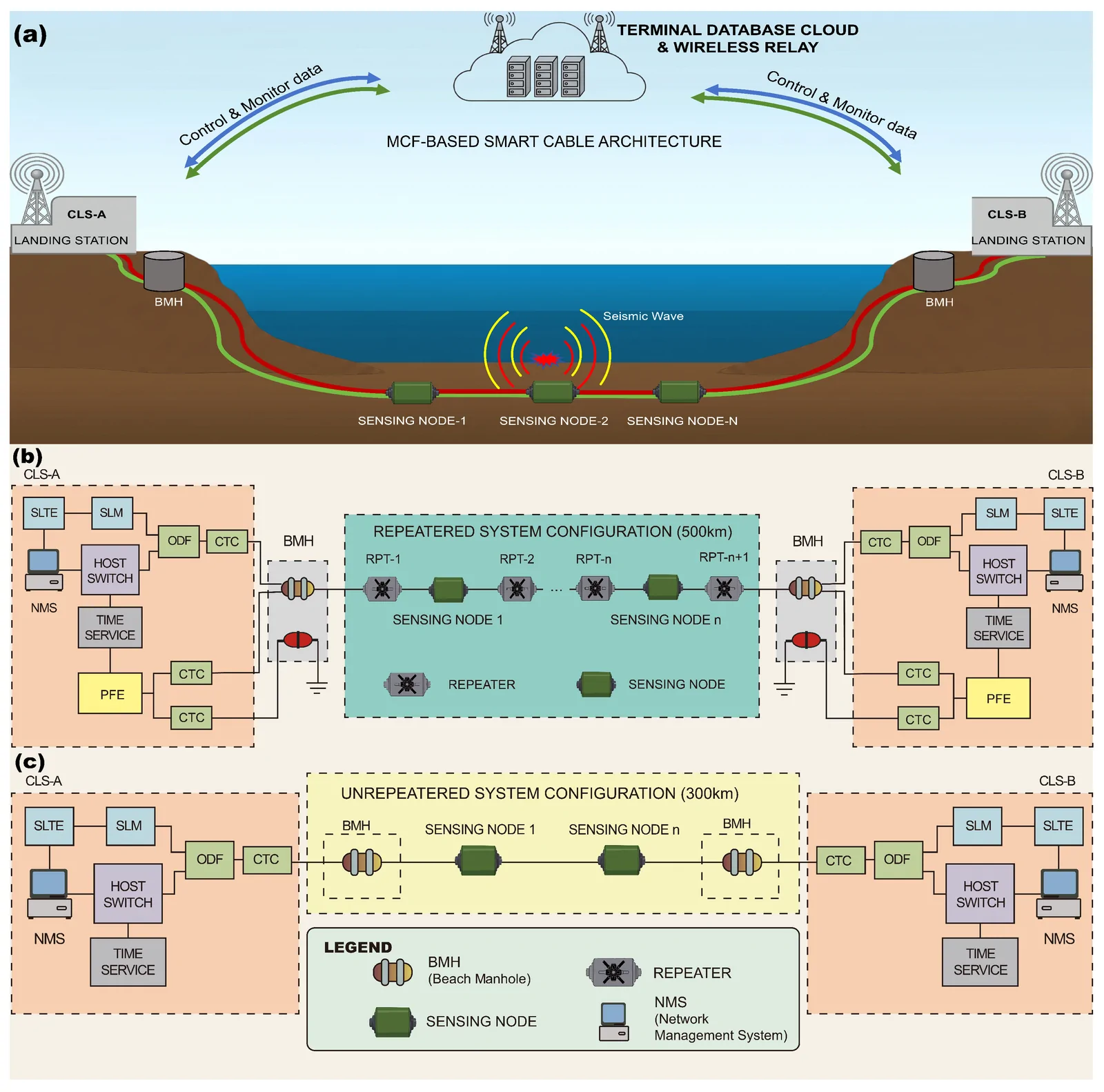
Conceptual architecture of the MCF-based smart cable system: (**a**) marine application scenario connecting two cable landing stations and discrete sensing nodes; (**b**) 500 km repeatered architecture with independent sensing nodes and communication repeaters; and (**c**) 300 km unrepeatered architecture.

**Figure 2:**
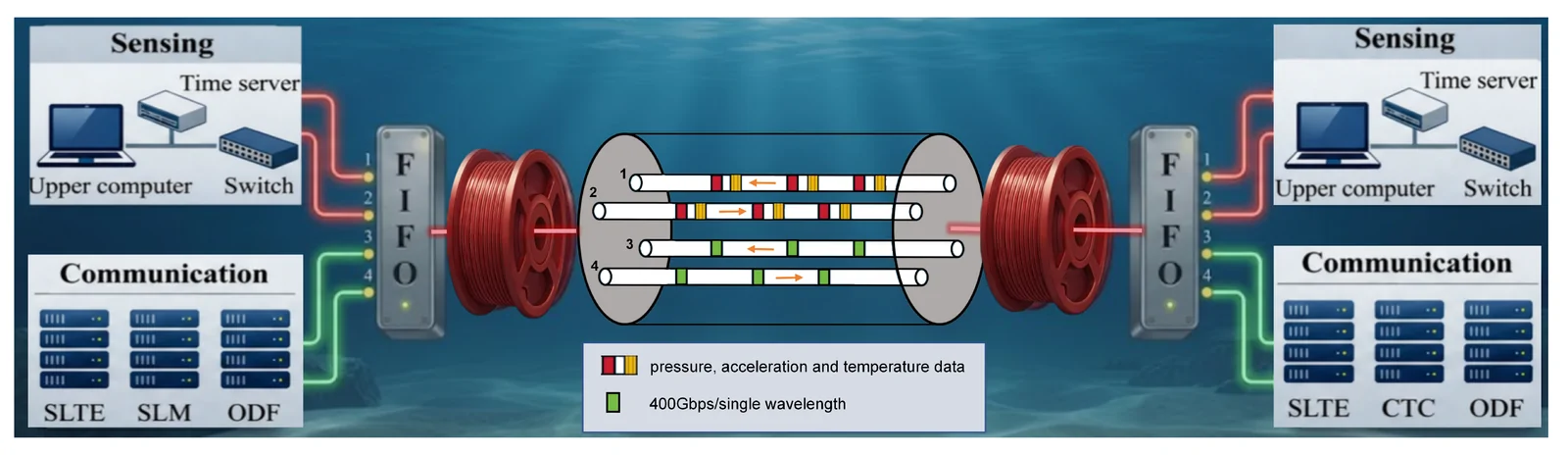
Core assignment and sensing-node access in the proposed MCF-based smart cable architecture. The communication and sensing-data functions use separate transmit/receive core pairs, while fan-in/fan-out devices provide access between the MCF and the corresponding equipment paths.

**Figure 3:**
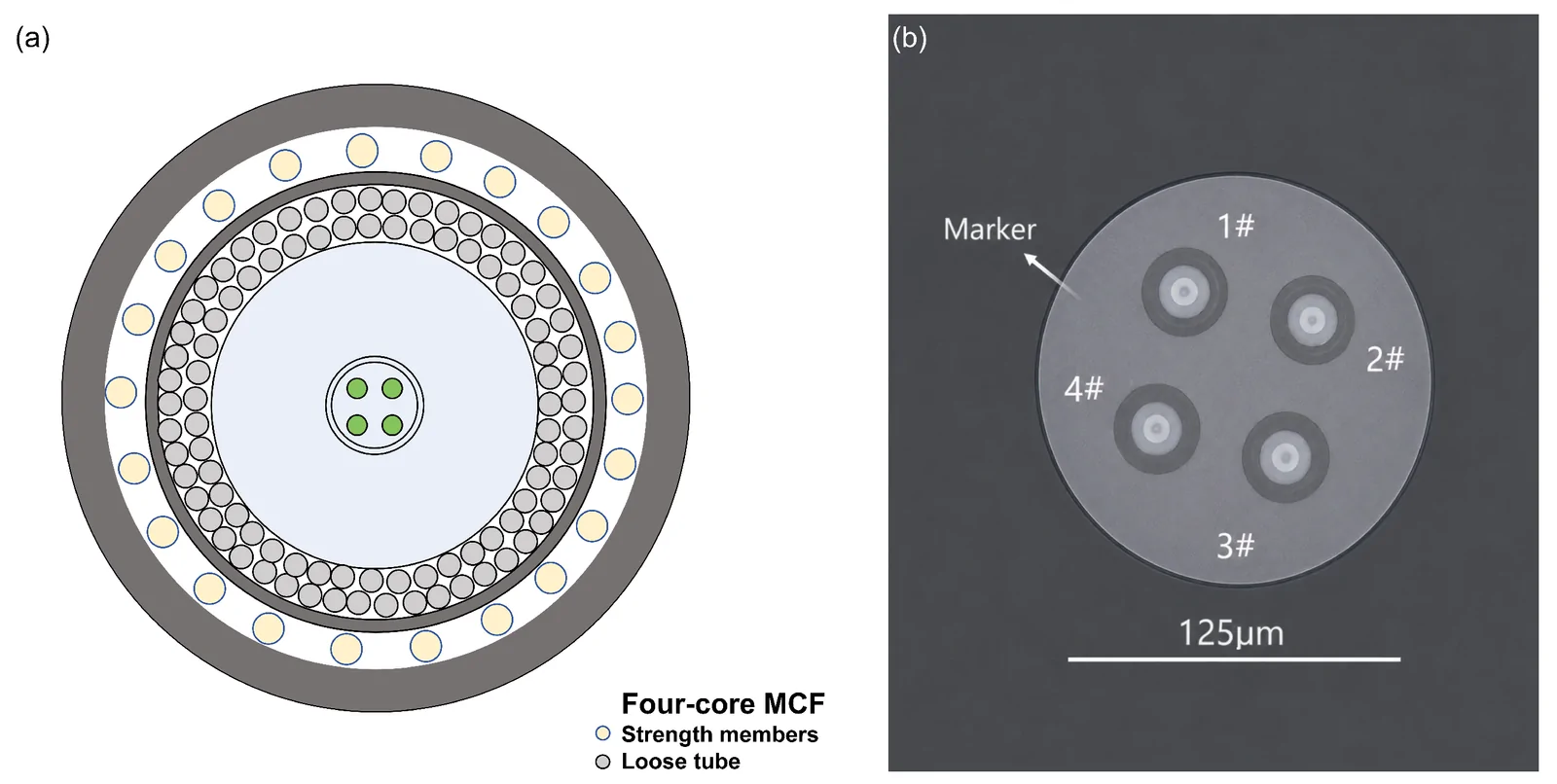
Representative views associated with the four-core MCF and its marine-cable integration: (**a**) cable cross-sectional arrangement and (**b**) four-core MCF end face. The four green circles in panel (**a**) denote the four cores of the MCF. The optical parameters used in the prototype analysis are fiber-level pre-cabling characterization data.

**Figure 4:**
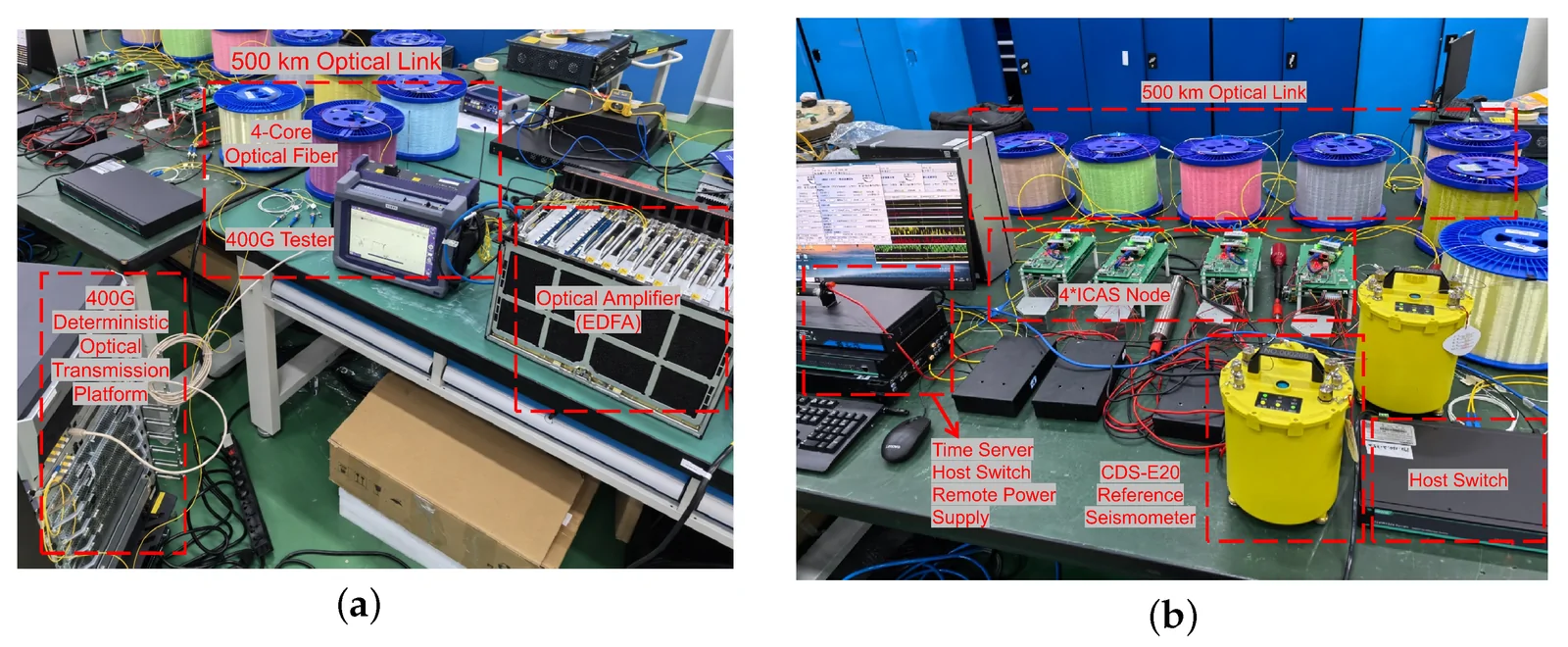
Laboratory realization of the smart cable prototype: (**a**) communication-side equipment, including the 400 Gbps transmission/test platform, optical amplification, and long-distance fiber link; and (**b**) sensing-side equipment, including sensing nodes, two shore-side host switches, the time server, remote-power equipment, and the CDS-E20 reference seismometer.

**Figure 5:**
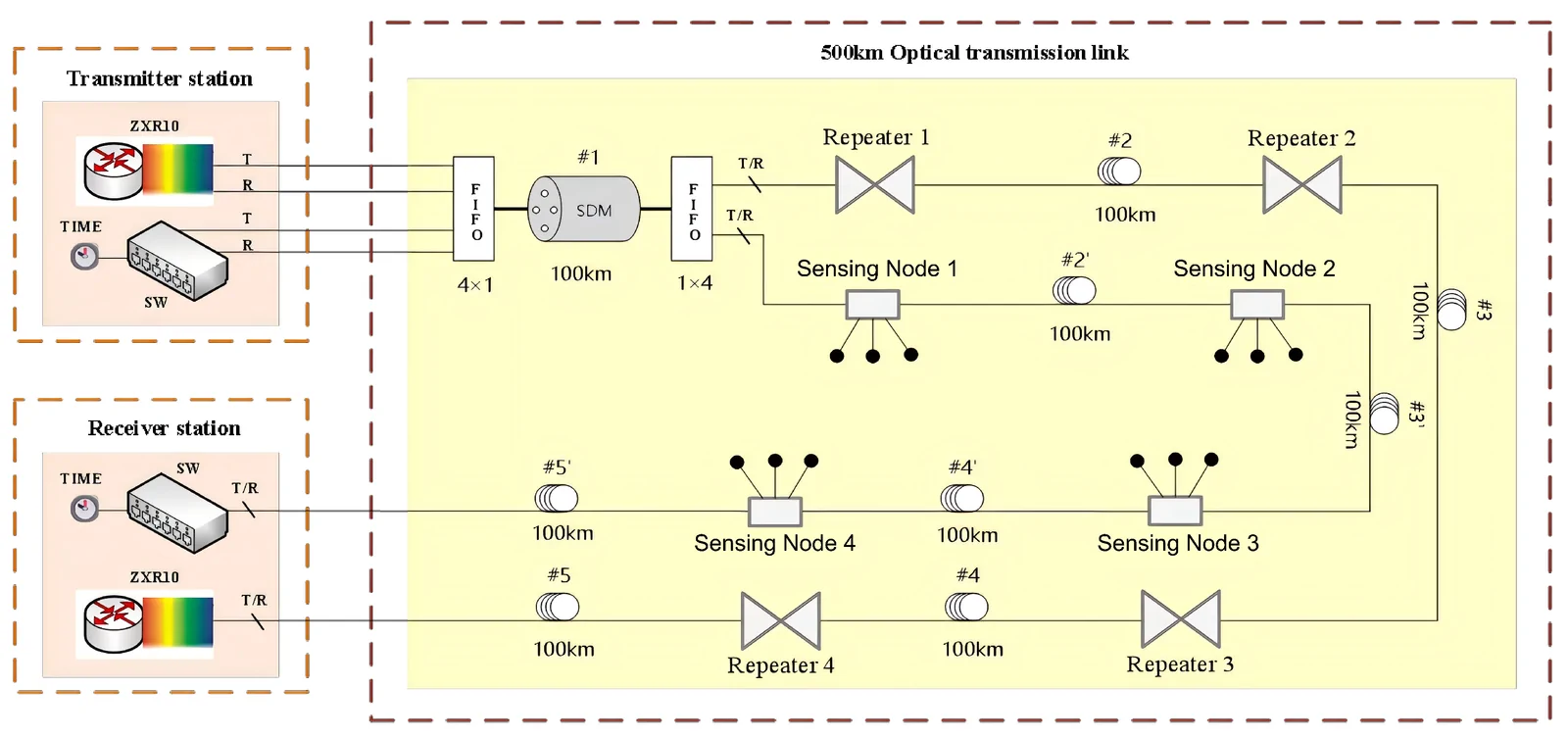
500 km repeatered laboratory configuration. The 100 km four-core MCF provides the spatial-routing section, and additional G.654.D SMF spans extend the total path. T/R denotes the separate transmit/receive core paths; ZXR10 denotes the 400 Gbps coherent transceiver platform, SW the sensing-data host switch, and FI/FO the fan-in/fan-out devices. The laboratory repeater blocks provide optical gain only and do not perform 3R regeneration.

**Figure 6:**
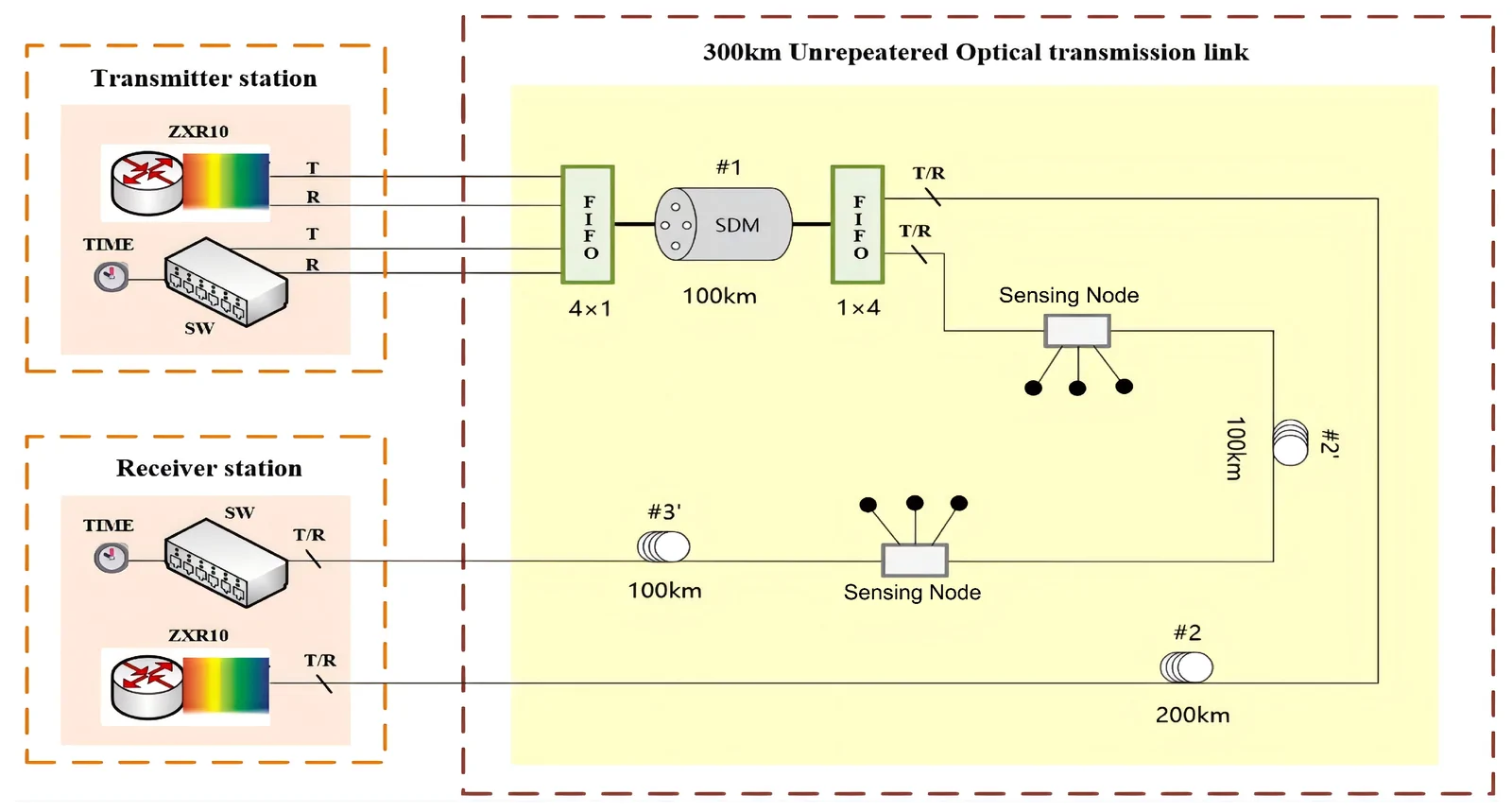
300 km unrepeatered trunk-and-branch laboratory configuration using the same 100 km four-core MCF spatial-routing section and additional G.654.D SMF spans. T/R denotes the separate transmit/receive core paths.

**Figure 7:**
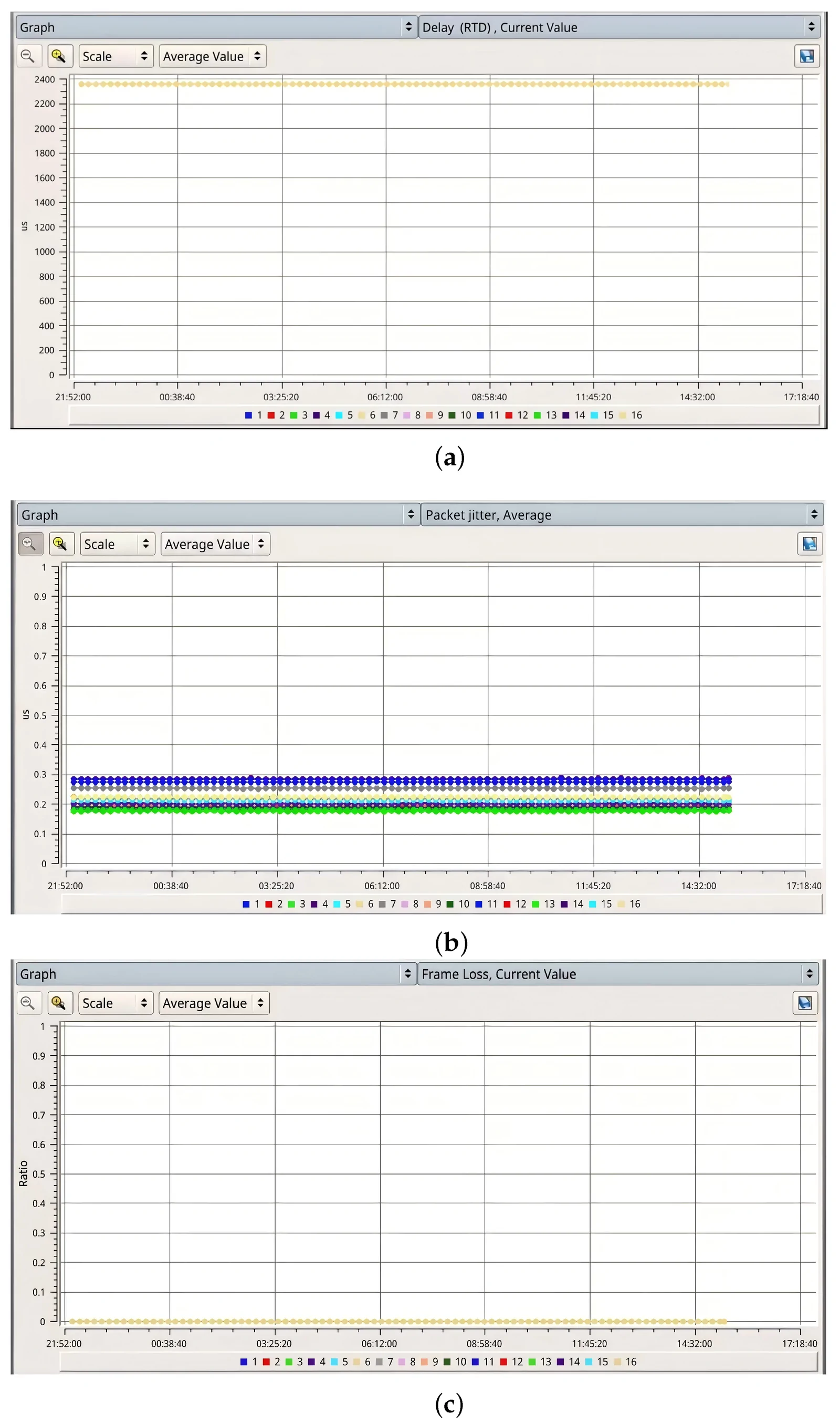
Communication performance at CH26 (1538.98 nm) under laboratory co-transmission conditions: (**a**) round-trip delay (RTD), (**b**) packet jitter, and (**c**) current frame-loss count.

**Figure 8:**
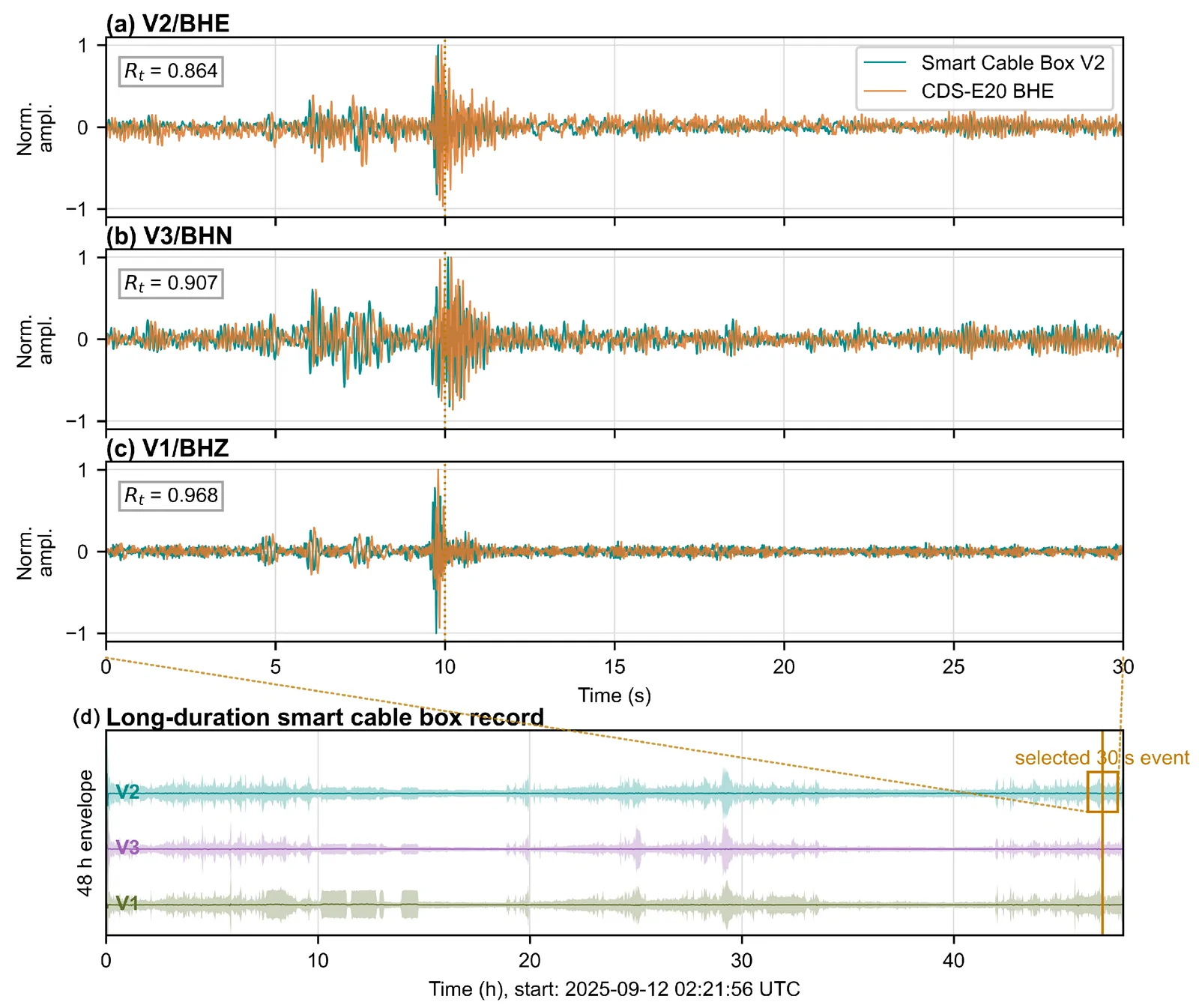
Time-domain velocity comparison between the sensing-node velocity channels and the CDS-E20 reference seismometer for the representative 30 s transient segment starting at 14 September 2025 01:23:10 UTC: (**a**) V2/BHE, (**b**) V3/BHN, (**c**) V1/BHZ, and (**d**) location of the selected 30 s segment within the long-duration record. The dashed guide lines indicate the selected 30 s interval in panel (**d**) corresponding to the waveform comparisons in panels (**a**–**c**). $R_{t}$ denotes the time-domain Pearson correlation coefficient.

**Figure 9:**
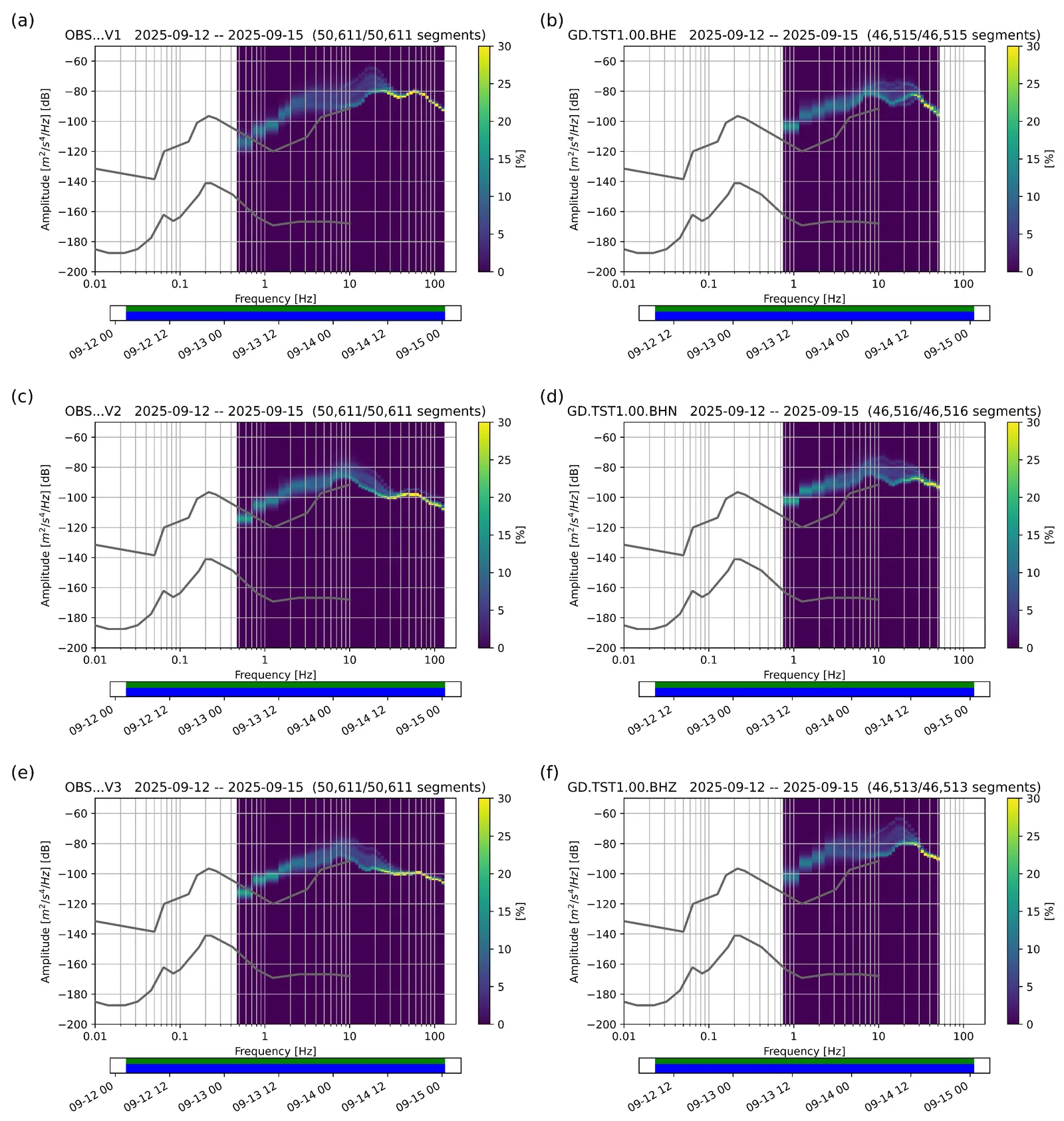
Long-duration velocity PPSD comparison calculated with 60 s windows and 50% overlap: (**a**) sensing-node V1, (**b**) CDS-E20 BHE, (**c**) sensing-node V2, (**d**) CDS-E20 BHN, (**e**) sensing-node V3, and (**f**) CDS-E20 BHZ. The color scale indicates the occurrence probability of spectral-power values, and the reference curves are the standard seismic noise models provided by ObsPy.

**Table 1:** Selected structural and optical characterization values from the final inspection record for the four-core MCF used in the prototype.

Parameter	Measured Value(s)	Relevance to the Prototype
Cladding diameter	124.73 μm	Common-cladding geometry.
Adjacent-core spacing	42.798, 42.981, 42.815, 42.833 μm	Spatial separation between neighboring cores.
Opposite-core spacing	60.550, 61.667 μm	Diagonal core separation.
Attenuation at 1550 nm	0.182 dB/km for all four reported measurements	Transmission-loss assessment.
Mode-field diameter at 1550 nm	10.73, 10.84, 10.79, 10.72 μm	Modal size of the transmission cores.
Cutoff wavelength	1443.25, 1448.56, 1443.47, 1448.32 nm	Single-mode operating characteristic.
Chromatic dispersion at 1550 nm	20.368, 20.377, 20.378, 20.390 ps/(nm·km)	C-band transmission dispersion.
Numerical aperture	0.12	Optical acceptance characteristic.
Macrobending loss at 1625 nm	0.05, 0.02, 0.06, 0.04 dB (Φ30 mm × 10 turns)	Measured bending sensitivity.
Inter-core crosstalk at 1625 nm	−53.1, −52.6, −51.2, −53.7 dB/100 km	Direct optical leakage between cores.
Group refractive index	1.4683 at 1550 nm; 1.4684 at 1625 nm	Group-propagation characteristic.

## Data Availability

The data supporting the findings of this study are available from the corresponding author upon reasonable request.
